# Navigating the policy landscape: a systematic mapping review of national access and benefit-sharing frameworks for pathogens

**DOI:** 10.3389/fpubh.2026.1908687

**Published:** 2026-09-04

**Authors:** Ruiyu Geng, Hong Yu

**Affiliations:** Institute of International Law, Wuhan University, Wuhan, China

**Keywords:** access and benefit-sharing, genetic resources, national policy, PABS system, pathogen, WHO pandemic agreement

## Abstract

**Background/objectives:**

Pathogen access and benefit-sharing (PABS) sits between two practical needs. Public health depends on rapid access to pathogen materials and sequence information, while countries also seek a fair share of the benefits arising from their use. Comparative evidence on the domestic rules governing this exchange remains limited. This study maps national PABS frameworks and examines what their variation may mean for the emerging WHO PABS System.

**Methods:**

This study conducted a systematic mapping and comparative analysis across all 193 United Nations Member States. International legal databases, official national sources, and targeted searches were used to identify relevant binding frameworks. Legally enforceable policies were identified, and data were extracted across the following four aspects of PABS legislation: (1) Resource Classification, (2) Access Protocols, (3) Benefit-Sharing, and (4) Regulatory Mechanism. Country-level sources, screening decisions, and coding rules are documented in the Supplementary materials.

**Results:**

Ninety-three countries had confirmed frameworks covering pathogen-related genetic resources, while three cases remained legally unclear. Restricted access and formal requirements for prior authorization, benefit-sharing, and compliance were common among confirmed frameworks. DSI was less settled. National approaches ranged from explicit inclusion or exclusion to silence, and the pattern differed markedly across regions. Contractual requirements and legal sanctions also varied.

**Conclusion:**

The national PABS landscape is fragmented rather than convergent. A future WHO PABS System will have to operate across different domestic approaches to authorization, documentation, sequence information, and benefit-sharing. More predictable interfaces between national systems may therefore be more workable than uniform national legislation.

## 1 Introduction

The history of human civilization is also a history of combating disease and disaster. Since the mid-20th century, pathogens have increasingly crossed national borders, with cross-border outbreaks repeatedly accompanied by debates over pathogen access and benefit-sharing (ABS) (1). Pandemics are not constrained by territorial boundaries, and every nation faces direct or indirect threats. Establishing a comprehensive system for pandemic prevention, preparedness, and response is critical to safeguarding public health and security. Advances in biomedicine and recurrent international public health emergencies have made pathogens vital “raw materials” for public health innovation and global common interests (2). These resources generate substantial economic and disease-control value while also becoming focal points of international rights disputes. The pharmaceutical industry’s ability to commercialize products derived from pathogens provided by nations has intensified divergences between developing and developed countries. Developing countries assert sovereign rights over pathogens and advocate prior informed consent (PIC) and benefit-sharing regimes. They emphasize binding commitments requiring developed nations to share benefits arising from the utilization of these biological and genetic resources. In contrast, developed nations frame pathogens as a “common heritage of humankind” and promote voluntary benefit-sharing mechanisms decoupled from pathogen access. This approach enables the unregulated acquisition of pathogen materials while retaining intellectual property protections for derived medical products (3). Existing mechanisms perpetuate structural imbalances favoring developed nations over developing countries, thereby exacerbating the PABS dilemma.

In the face of global public health challenges, ensuring equitable access to pathogens and the fair sharing of benefits is a critical issue that the academic and international communities must address together. Given the legal status and regulatory uncertainties surrounding pathogens, this study approaches the problem in two linked steps. It first reviews the main international arrangements that have shaped pathogen access, including the Convention on Biological Diversity (CBD), the Nagoya Protocol on Access to Genetic Resources and the Fair and Equitable Sharing of Benefits Arising from their Utilization to the Convention on Biological Diversity (Nagoya Protocol), the Pandemic Influenza Preparedness Framework for the Sharing of Influenza Viruses and Access to Vaccines and Other Benefits (PIP Framework), and the WHO Pandemic Agreement. It then compares national PABS policies across all 193 UN Member States using the four analytical domains established in the study design. Earlier research has examined particular jurisdictions and individual elements of PABS, but transparent comparison across the full UN membership remains limited. The mapping shows where national rules share common features, where they differ, and where legal scope remains uncertain. It also provides a domestic baseline for assessing the future WHO PABS System. The mapping therefore provides a foundation for understanding the current global PABS landscape and an evidence base for future bilateral, regional, and global negotiations.

### 1.1 Origins and early development of the controversy

Effective pathogen access and benefit-sharing constitute foundations for infectious disease research and are central to the development of innovative drugs and vaccines. The international community has recognized the significance of pathogen access and sharing since the 20th century and has undertaken substantial institutional development in this field. In the first half of the 20th century, following the development of the WHO-coordinated International Network of Influenza Laboratories, the Global Influenza Surveillance and Response System (GISRS) (4) was formally established in 1952. The system primarily relies on the open sharing of influenza viruses worldwide to monitor seasonal influenza transmission and detect emerging pandemic strains (5). Although the initial volume of pathogen samples exchanged within GISRS remained relatively limited, the initiative represented an organized and systematic early practice of global pathogen sharing.

However, against this backdrop, the international community had yet to establish a comprehensive normative framework or supervisory system governing PABS. This regulatory gap occasionally enabled foreign scientists to obtain pathogen samples without proper national authorization through informal research collaborations. Debate over biological resources later shifted as biodiversity-rich countries pressed for greater national control. These collective efforts ultimately culminated in the adoption of the CBD during the 1990s, marking a pivotal development in global biological resource governance (6). In 2002, the sixth meeting of the Conference of the Parties (COP 6) to the CBD adopted the voluntary Bonn Guidelines, which provide general guidance on access to genetic resources and the fair and equitable sharing of benefits arising from their utilization. Although not pathogen-specific or legally binding, the Guidelines provided an early procedural framework relevant to pathogen-related genetic resources within the scope of the CBD (7). Relevant provisions include Article 15 (1) on national sovereignty over genetic resources, Article 15 (2) on access for environmentally sustainable applications, Article 15 (5) on prior informed consent, and Article 15 (7) on equitable benefit-sharing, together with Articles 16–19 on technology transfer and international cooperation (8). These rules were developed in biodiversity law, but they can affect pathogens when domestic ABS legislation brings pathogen-related genetic resources within its scope.

Public health law developed along a parallel track. With the advent of the 21st century, global attention to PABS has intensified. In this context, the World Health Assembly (WHA) has adopted multiple resolutions urging Member States to facilitate the rapid and transparent sharing of clinical samples, viral strains, and biological materials through GISRS and collaborating centers during influenza outbreaks, particularly when novel influenza strains emerge. This practice has not only enhanced international information exchange but also strengthened global public health security (9). The International Health Regulations (2005) (IHR), with particular reference to Articles 6 and 7 concerning information and public health response, set out provisions for the exchange of information relating to public health events of international significance. Nevertheless, controversy remains over whether the “public health information” required to be reported under the IHR encompasses pathogen information, and the issue of pathogen access and sharing has not been resolved (10). The global health security framework centered on the IHR focuses on safeguarding the interests of developed countries and does not provide timely, specific institutional arrangements to address imbalances in pathogen access and benefit-sharing. Through resolutions adopted since 2006 on avian influenza and implementation of the IHR, the WHA has also “recognized States’ sovereign rights over their biological genetic resources” (11). Persistent interpretive divergences regarding these provisions have hindered consensus-building. To safeguard the interests of developing nations facing substantial influenza burdens and limited response capacity, WHO has actively engaged Member States in developing frameworks for pandemic influenza preparedness since 2007.

From the perspective of international law, sovereignty remains a foundational principle of the international legal order. Indonesia’s decision in late 2006 and early 2007 to withhold H5N1 influenza virus samples from the WHO system, while invoking the CBD and concerns over unequal access to vaccines, constituted an assertion of sovereign authority over access to biological resources located within its territory. The dispute brought developing countries’ demands for equitable participation in global public health governance to international attention and gave prominence to the contested concept of “viral sovereignty” (12, 13). Subsequent controversies concerning access to pathogen samples and sequence information during outbreaks of MERS-CoV, H7N9 avian influenza, Ebola, and Zika further exposed persistent tensions between the rapid sharing of pathogens and the equitable distribution of resulting benefits (14).

Against this broader background, the tenth meeting of the Conference of the Parties (COP 10) to the CBD adopted the Nagoya Protocol in 2010 as a supplementary agreement to the Convention (15). The Protocol operationalizes the CBD’s objective by establishing a framework for the fair and equitable sharing of benefits arising from the utilization of genetic resources. Where required by the domestic access and benefit-sharing legislation or regulatory requirements of the provider Party, access to genetic resources is subject to prior informed consent. Benefits arising from their utilization must be shared on mutually agreed terms (16, 17). The CBD and the Nagoya Protocol recognize the sovereign rights of States over their natural resources and affirm that the authority to determine access to genetic resources rests with national governments and is subject to domestic legislation.

The application of the Nagoya Protocol to pathogens has nevertheless generated operational complexity. Differences among national access and benefit-sharing requirements may create administrative burdens and legal uncertainty that can delay or restrict the cross-border transfer of pathogen samples. At the same time, advances in sequencing technologies have enabled researchers to make increasing use of DSI without transferring physical specimens. Because the Nagoya Protocol does not expressly address DSI, its application to such information has remained contested. The establishment of the CBD multilateral mechanism for benefit-sharing from the use of DSI and the Cali Fund has partially addressed this gap. However, important questions remain concerning implementation and the relationship between these developments, the Nagoya Protocol’s bilateral framework, and the emerging WHO PABS System (18–20).

### 1.2 Major public health emergencies drive regulatory evolution

Influenza viruses are highly mutable and can trigger global pandemics, posing substantial threats to public health. Given their pathogenicity and cross-border transmission risks, global demand for the sharing of influenza virus samples has intensified. In May 2007, the 60th WHA adopted its first resolution on pandemic influenza preparedness, addressing the sharing of viral specimens and access to vaccines and other benefits (21). Following protracted negotiations that culminated in consensus among Member States, the 64th WHA endorsed the PIP Framework in 2011. The Framework established norms for international cooperation in global influenza surveillance and response, facilitating the exchange of influenza viruses with pandemic potential, vaccines, and associated benefits (22, 23).

Transfers of PIP Biological Materials are governed by two Standard Material Transfer Agreements. SMTA1 governs transfers among laboratories within GISRS, whereas SMTA2 is a legally binding agreement between WHO and an entity outside GISRS that receives PIP Biological Materials from a GISRS laboratory. Genetic sequence data are also addressed within the broader PIP Framework, but they are not included in the definition of PIP Biological Materials and therefore do not follow the same material-transfer pathway (24–26). The Framework’s benefit-sharing system operates principally through SMTA2 commitments and the Partnership Contribution. SMTA2 commitments may include the provision of vaccines, antiviral medicines, diagnostic products, technology transfer, licensing, and other pandemic-response products or services, while the Partnership Contribution supports activities such as laboratory and surveillance capacity-building, regulatory capacity-building, and other preparedness measures (23).

The PIP Framework remains important because it links the sharing of H5N1 and other influenza viruses with human pandemic potential to concrete benefit-sharing arrangements. It also distinguishes between physical PIP Biological Materials and genetic sequence data. Only the former fall within the definition of PIP Biological Materials and are transferred through the SMTA mechanism, while genetic sequence data are addressed separately within the broader Framework (24–26). Its scope is narrow, however, and it does not resolve the broader questions arising under the CBD and the Nagoya Protocol. It also demonstrates the importance of predictability. Laboratories and public health authorities require rapid access to influenza viruses during an outbreak, while provider countries need confidence that benefit-sharing commitments will be meaningful and enforceable. The Framework does not resolve every question concerning genetic sequence data or the relationship between specialized public health arrangements and general ABS law. Even so, it provides an important institutional precedent for ongoing negotiations on the PABS Annex and the development of the WHO PABS System (27).

These developments not only reflect respect for sovereign rights over genetic resources but also demonstrate international solidarity in addressing global health crises. Implementation of the PIP Framework has reinforced multilateral collaboration in influenza surveillance, data sharing, and vaccine R&D, thereby providing robust legal safeguards against pandemic threats. The evolution of these governance mechanisms serves as both a complement to the CBD and its Nagoya Protocol and a critical refinement of PABS rules in the context of major public health emergencies. This progression underscores the need to reconcile State sovereignty with collective action in balancing biosecurity imperatives and global health equity. Since the PIP Framework was established, global health experts have persistently advocated enhanced governance of PABS during outbreaks. Yet PABS remains an area of insufficient oversight within global health governance. Although the PIP Framework operationalizes specific sharing mechanisms, it has not reconciled tensions arising from assertions of viral sovereignty, leaving unresolved challenges in balancing national rights over pathogens with global health collaboration.

The scope of the PIP Framework is limited to H5N1 and other influenza viruses with human pandemic potential. Genetic sequence data fall within the broader PIP architecture but are not included in the definition of PIP Biological Materials and therefore do not automatically follow the same SMTA-based access and benefit-sharing pathway. As a non-treaty instrument adopted by the World Health Assembly, the Framework does not impose a treaty-level obligation on Member States to share virus samples. Its virus-sharing provisions generally use non-mandatory language. Within GISRS, however, SMTA1 establishes binding terms governing transfers among participating laboratories, while the 2017 Operational Guidance provides further direction on the selection and shipment of influenza viruses with human pandemic potential. Because the Guidance is not legally binding and does not establish uniformly enforceable deadlines, implementation may remain uneven. Virus sharing may also be delayed by national approval procedures and additional bilateral ABS requirements (21). The Framework operates through two Standard Material Transfer Agreements together with the Partnership Contribution. SMTA1 governs transfers of PIP Biological Materials among GISRS laboratories. SMTA2 is a legally binding agreement between WHO and a non-GISRS entity receiving such materials, including manufacturers and research institutions. Despite these mechanisms, the Framework remains limited by its narrow pathogen scope, its incomplete treatment of genetic sequence data, and its reliance on contractual arrangements for benefit-sharing.

The legal status of the PIP Framework under Article 4 (4) of the Nagoya Protocol remains subject to differing approaches. The World Health Assembly has emphasized its function as a specialized international instrument, and several jurisdictions recognize it as a specialized international ABS instrument. However, no uniform recognition process has yet been agreed by the Parties to the Nagoya Protocol. Consequently, the extent to which the PIP Framework displaces bilateral ABS requirements may vary across jurisdictions. It is therefore best understood as a compromise negotiated by Member States following disputes over H5N1 virus sharing, intended to balance rapid access to influenza viruses, equitable benefit-sharing, and global public health needs.

The COVID-19 pandemic again demonstrated how rapidly a novel pathogen can spread across borders and how strongly public health research and response depend on the timely sharing of pathogen samples and genetic sequence data (28). The crisis exposed major deficiencies in global preparedness for public health emergencies and reaffirmed the importance of timely sharing of pathogen samples and genetic sequence data among laboratories, governments, and relevant private entities. Such cooperation proved indispensable for pandemic surveillance, risk assessment, vaccine research and development, and the development of diagnostics and therapeutics (26, 28). In response, WHO launched several initiatives to strengthen pathogen sharing and surveillance. The WHO BioHub System was launched in November 2020 as a global platform for the voluntary sharing of biological materials with epidemic or pandemic potential and to facilitate the development of medical countermeasures (23). In May 2021, WHO and the Swiss Confederation established the first WHO BioHub Facility at the Spiez Laboratory. Its pilot phase used SARS-CoV-2 variants to develop operational arrangements for the rapid and equitable sharing of pathogen materials while seeking to complement existing laboratory networks and repositories (24, 29, 30). In May 2023, WHO launched the International Pathogen Surveillance Network to strengthen pathogen genomic surveillance, connect relevant actors, accelerate the detection and assessment of infectious disease threats, and promote international collaboration (25, 31). Its effectiveness nevertheless depends on sustained political cooperation, adequate technical capacity, and sufficient funding.

These initiatives strengthened the institutional infrastructure for international cooperation, but they did not establish a comprehensive multilateral legal framework governing access to pathogen materials, the conditions attached to their use, and the sharing of resulting benefits. The COVID-19 pandemic also exposed substantial inequalities in access to vaccines, diagnostics, and therapeutics (32). More broadly, it revealed continuing fragmentation and regulatory gaps in the international legal architecture for pathogen access and benefit-sharing. Existing instruments did not provide a comprehensive mechanism capable of simultaneously ensuring rapid pathogen sharing and the equitable distribution of benefits arising from research and development. These concerns accelerated international negotiations on reforming the global legal framework for pandemic prevention, preparedness, and response (27, 33).

### 1.3 New pathways for global health governance in the post-pandemic era

The COVID-19 pandemic exposed a persistent imbalance between the rapid international sharing of pathogen materials and sequence information and the unequal global distribution of the medical countermeasures developed through scientific research. The early sharing of SARS-CoV-2 sequence information enabled research networks to accelerate the development of vaccines, therapeutics, and diagnostics, yet access to these products remained highly uneven across countries. This experience intensified debate over whether rapid scientific exchange could be accompanied by more predictable and equitable arrangements for sharing the benefits arising from the use of pathogen materials and sequence information (32, 34).

On 20 May 2025, the Seventy-eighth World Health Assembly adopted the WHO Pandemic Agreement after more than 3 years of negotiations. Article 12 recognizes both the sovereign rights of States over their biological resources and the importance of collective action to mitigate public health risks. On this basis, it establishes the multilateral WHO Pathogen Access and Benefit-Sharing System. The provision places two objectives on an equal footing: the rapid and timely sharing of PABS Materials and Sequence Information and the rapid, timely, fair, and equitable sharing of monetary and non-monetary benefits arising from their sharing or utilization (33, 35, 36). Such benefits include monetary contributions as well as access to vaccines, therapeutics, and diagnostics. During a pandemic emergency, the distribution of relevant medical countermeasures made available through the PABS System is to be based on public health risk and need, with particular attention to the needs of developing countries (35, 36). As a multilateral mechanism, the PABS System therefore moves beyond a purely bilateral, transaction-by-transaction model in which access to each pathogen resource is matched with a corresponding benefit for an individual provider country.

Although Article 12 establishes the PABS System and its principal objectives, many of the rules required for its operation remain to be developed in the PABS Annex. Resolution WHA78.1 established an open-ended Intergovernmental Working Group to draft and negotiate the Annex. The outcome was initially expected to be submitted to the Seventy-ninth World Health Assembly in 2026. As negotiations were not completed, Member States subsequently agreed that the outcome should be submitted to the Eightieth World Health Assembly in May 2027 or, if the text is ready earlier, to a dedicated special session of the Health Assembly in 2026 (37, 38). Negotiations continued through the seventh meeting of the Intergovernmental Working Group in July 2026, but the Annex remained unfinished as of the study cutoff in August 2026. Adoption of the Annex is required before the WHO Pandemic Agreement can open for signature.

The negotiations have addressed a broad range of legal and operational questions, including the definitions and scope of pathogens with pandemic potential and PABS Materials and Sequence Information, modalities and conditions of access, traceability and access to sequence information, the identification or registration of participants and users, contractual arrangements, monetary and non-monetary benefit-sharing, biosafety and biosecurity, data protection, intellectual property, and the administration and coordination of the PABS System by WHO (37, 39–42). These questions are particularly important because the international PABS System will interact with national, regional, and other international ABS arrangements rather than operate in isolation. Article 12 already requires the PABS Instrument to be complementary to and non-duplicative of the PIP Framework and other relevant international ABS instruments where applicable. It also envisages that Parties will review and, as they deem appropriate, align national or regional ABS measures applicable to PABS Materials and Sequence Information so that measures that are contrary to, inconsistent with, or duplicative of the PABS Instrument are not applied once the PABS System becomes operational (36, 43). The considerable differences among existing national rules on authorization, DSI, contractual requirements, benefit-sharing, compliance, and sanctions therefore constitute an important part of the legal environment in which the future PABS System will operate.

A related development under the Convention on Biological Diversity provides a useful, although distinct, point of comparison. Decision 15/9 established a multilateral mechanism for the fair and equitable sharing of benefits from the use of digital sequence information on genetic resources. Decision 16/2, adopted at COP16, subsequently set out modalities for operationalizing that mechanism and named its global fund the Cali Fund (44). Under these modalities, qualifying commercial entities that directly or indirectly benefit from the use of DSI and exceed specified size thresholds should contribute, as an indicative rate, 1 percent of their profits or 0.1 percent of their revenue to the Fund (44, 45). Where appropriate and subject to national circumstances and legislation, at least half of the Fund’s resources should support the self-identified needs of indigenous peoples and local communities, including women and youth within those communities (45). The Cali Fund is not a pathogen-specific mechanism and operates under a different legal and institutional framework. Its relevance to the present discussion lies instead in its use of a multilateral approach to benefit-sharing from sequence information without linking every instance of use to a separate bilateral transaction. Whether, and to what extent, a comparable approach would be appropriate for pathogen-related sequence information under the PABS System remains a separate question.

PABS consequently sits at the intersection of global public health, biodiversity law, intellectual property, technology transfer, research cooperation, and distributive equity. States approach these issues from different legal, economic, scientific, and public health perspectives, and the remaining differences cannot be reduced to a simple divide between developed and developing countries (36–38). The WHO Pandemic Agreement establishes a common institutional framework but does not eliminate the diversity of the national legal systems with which the eventual PABS rules will need to interact. Against this background, the present study systematically maps national rules relevant to Pathogen-Related Genetic Resources Access and Benefit-Sharing across all 193 UN Member States. It does not seek to prescribe a single legal model for PABS. Rather, it identifies areas of convergence and divergence among existing national approaches and provides an empirical baseline for assessing how future international rules may interact with domestic and regional ABS measures. In this respect, the country-level mapping helps locate the legal points at which greater consistency, interoperability, or clarification may be required while preserving legitimate differences among national regulatory systems.

## 2 Materials and methods

### 2.1 Scope determination and national coverage methodology

**Figure 1:**
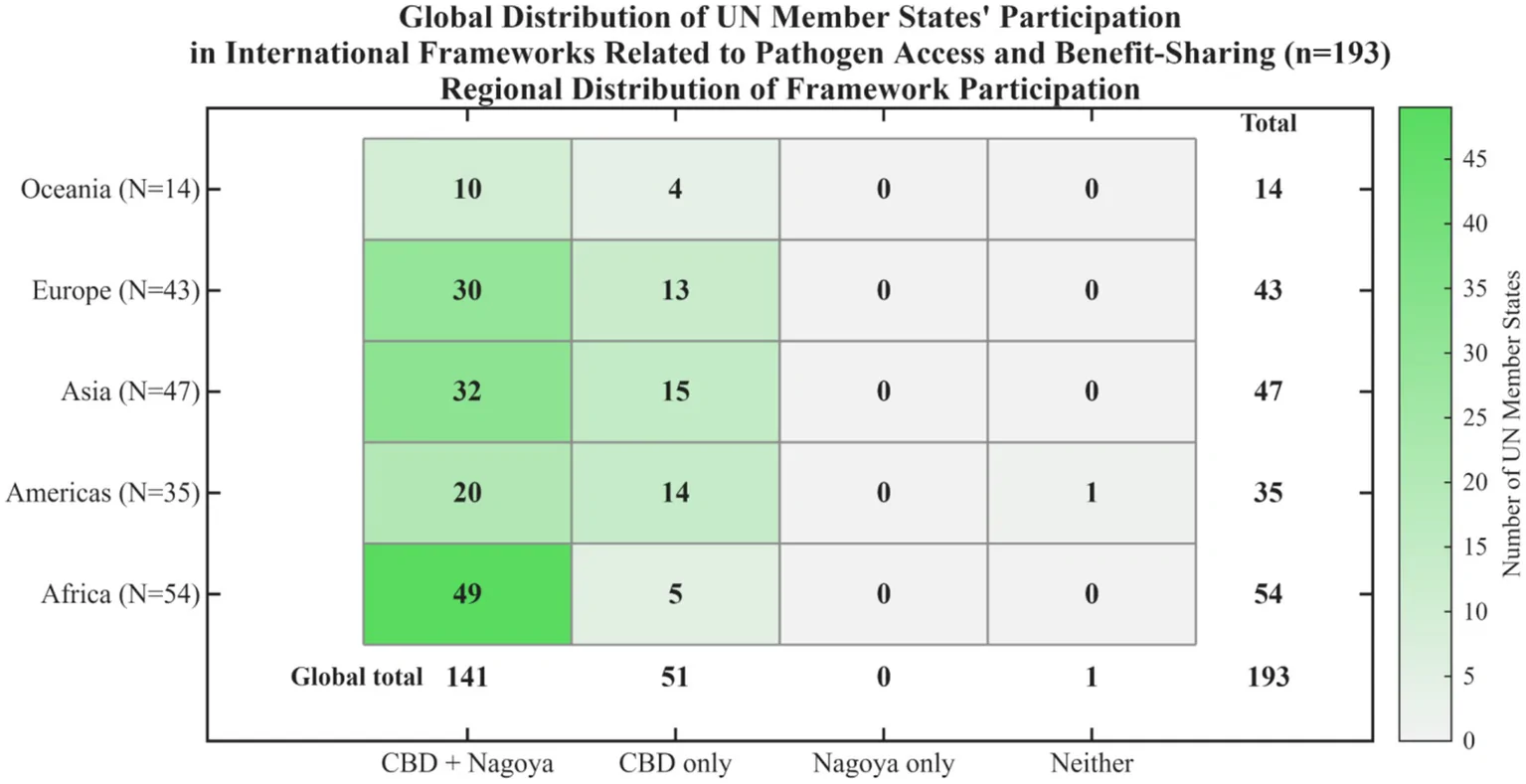
Global distribution of UN member states’ participation in international frameworks related to pathogen access and benefit-sharing.

This study examined national policies relevant to PABS in all 193 UN Member States. Membership in the CBD or the Nagoya Protocol was recorded as context but was not used to decide whether domestic law covered pathogens. The country was the principal unit of analysis. Figure 1 provides the treaty participation context before the analysis turns to domestic PABS coverage.

Treaty status was assessed as of August 2026. At the study cutoff, 192 of the 193 UN Member States were Parties to the CBD, while the United States, which has signed but not ratified the Convention, remained the only UN Member State that was not a Party. Among the same 193 States, 141 were Parties to both the CBD and the Nagoya Protocol, 51 were Parties to the CBD but not the Nagoya Protocol, and the United States was a Party to neither instrument (46, 47). Armenia had deposited its instrument of accession to the Nagoya Protocol on 8 June 2026, but the Protocol would not enter into force for Armenia until 6 September 2026. It was therefore classified as a non-Party to the Nagoya Protocol in the August 2026 study snapshot. The PIP Framework is not an international treaty and was therefore not included in this treaty participation matrix. It remains relevant to the analysis as a specialized framework for the sharing of influenza viruses with human pandemic potential and associated benefit-sharing within the WHO Global Influenza Surveillance and Response System (GISRS) (23, 48).

**Figure 2:**
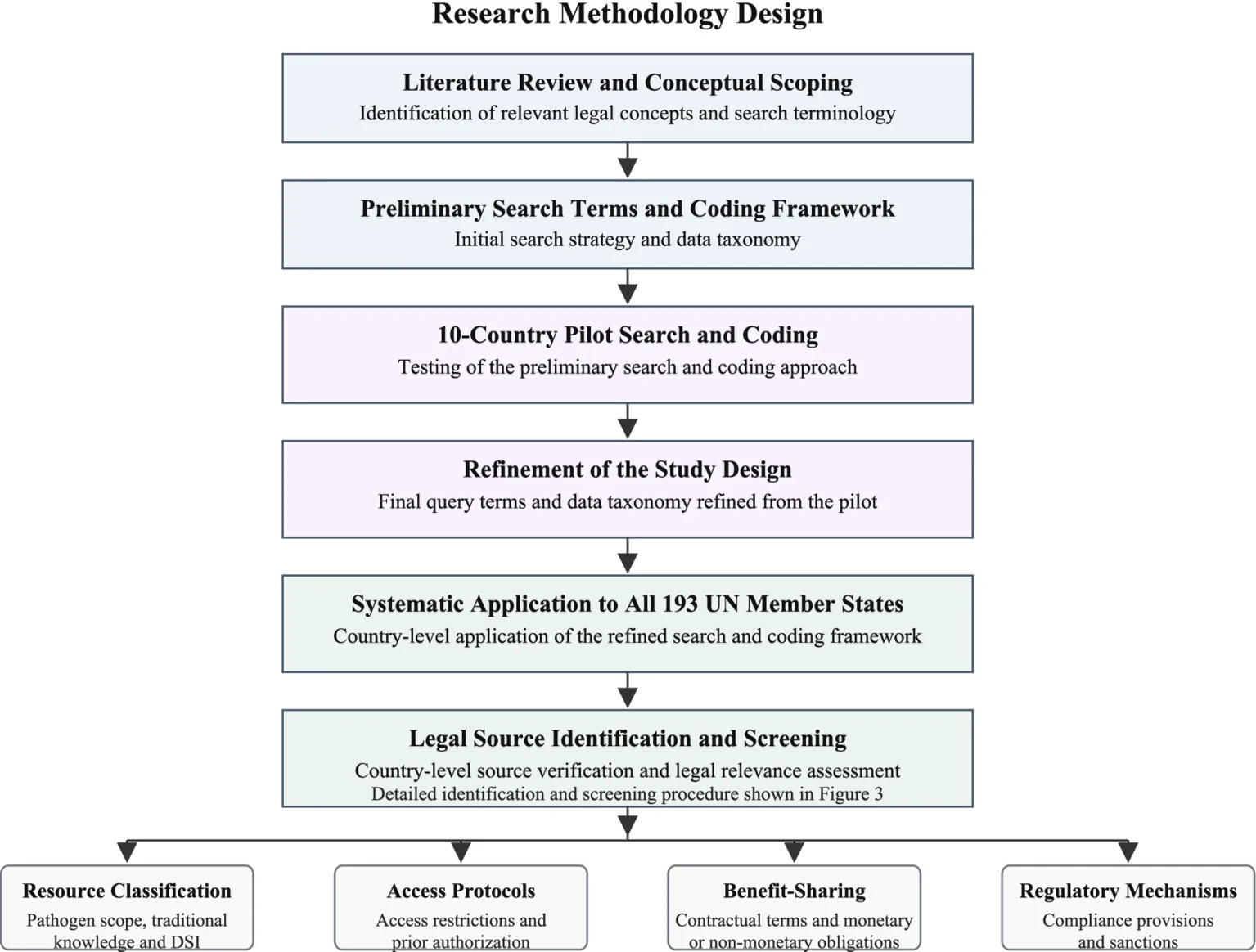
Research methodology design.

Figure 2 summarizes the sequence used to move from the preliminary search design to the final 193-country analysis. Data collection followed the approach set out in the original study design. We first reviewed the literature to identify search terms and legal concepts, then tested the search and coding approach in 10 countries. That exercise was used to refine the final query terms and data taxonomy. The retained project files do not contain a complete country-by-country record of the pilot. For that reason, the reproducibility materials focus on the fully disclosed 193-country dataset rather than reconstructing pilot details that were not preserved. The refined search terms and coding framework were then applied across all UN Member States.

### 2.2 Policy relevance determination

The search proceeded in stages. We began with international legal resources that contain ABS or genetic resource measures, including the ABS Clearing House, WIPO Lex and FAOLEX. We then checked official national legislation databases, gazettes and competent authority sources where these were available (49–52). Targeted web searches were used to locate authoritative records, not to replace legal verification.

**Figure 3:**
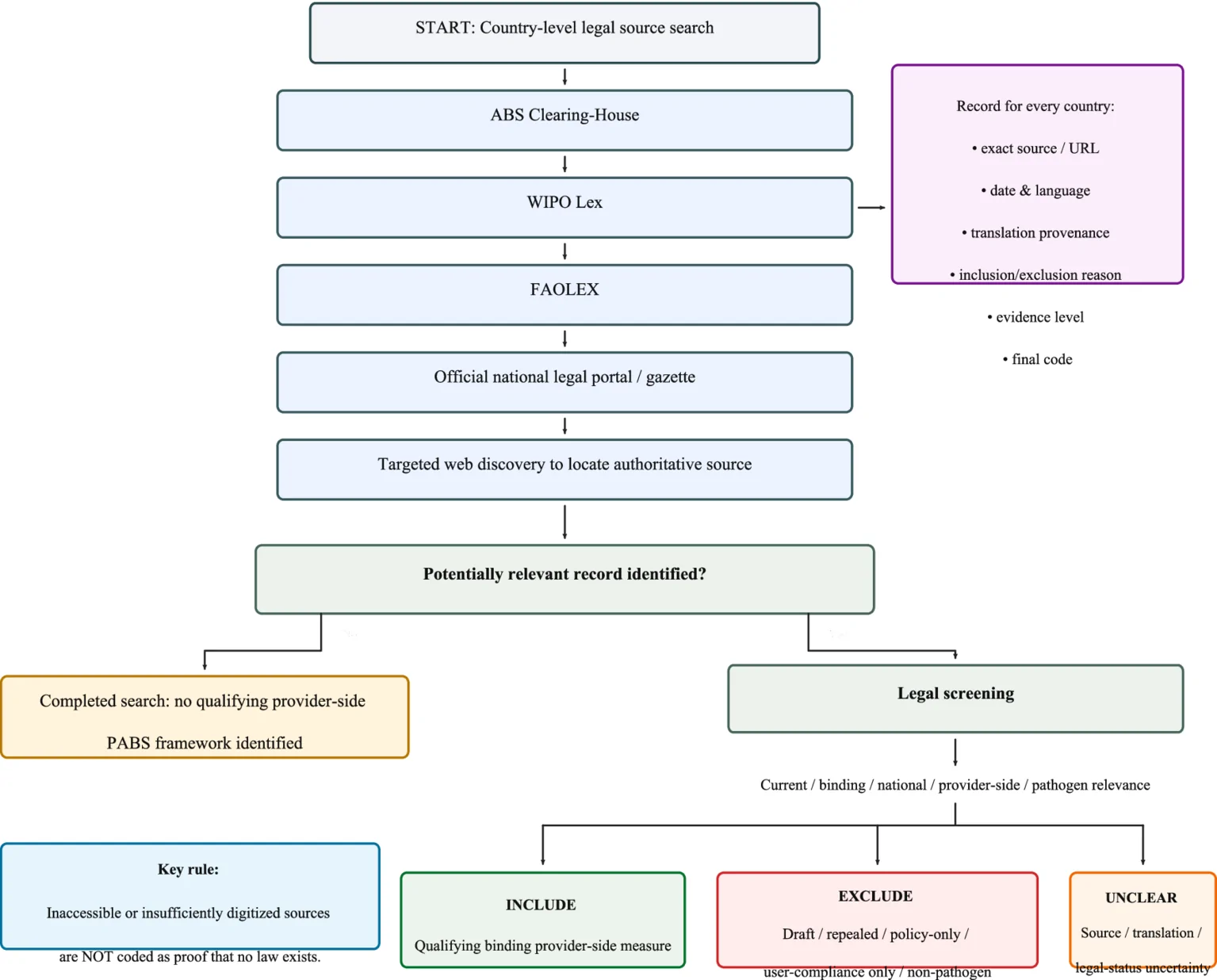
Policy identification and collection process.

Figure 3 shows how potentially relevant legal records moved from source identification through screening to inclusion, exclusion or unresolved status. Potential records were screened for legal status, national applicability, current validity, provider-side relevance and pathogen scope. Drafts, proposals and repealed instruments were excluded. Policy documents that did not create a binding legal requirement were also left outside the primary dataset, as were user compliance measures that did not establish provider-side access rules. An inaccessible or poorly digitized national source was recorded as a source limitation. It was not taken as proof that no law existed. When legal scope or source status could not be resolved, the country remained unclear rather than being forced into a binary category. Supplementary Table S2 reports the country-level search outcomes and the reasons for exclusion or uncertainty (53).

### 2.3 Database creation

Potentially relevant measures were first screened for legal force and current validity. We then read the retained texts in full to determine whether their operative scope reached pathogen-related genetic resources. Data were extracted using the four dimensions applied throughout the study. These were Resource Classification, Access Protocols, Benefit-Sharing and Regulatory Mechanisms (54).

Each retained measure was coded using the same framework. Resource Classification covers pathogen scope, traditional knowledge and DSI. Access Protocols cover restricted or unrestricted access and PIC or an equivalent prior authorization. Benefit-Sharing covers contractual terms and monetary or non-monetary obligations. Regulatory Mechanisms cover compliance provisions and sanctions. Official or intergovernmental English versions were used for non-English sources when available. Language and translation provenance are recorded in Supplementary Dataset S1 where they could be recovered. Records without reliable translation provenance are marked NR rather than inferred.

The final database contains one analytical record for each of the 193 UN Member States. Each record preserves the country identifier, region, final legal classification, source metadata, coding outcome and evidence notes. Supplementary Dataset S1 provides the country-level legal corpus and final codes. Supplementary Table S2 records search, screening and uncertainty outcomes. Supplementary Methods S3 contains the coding manual and search protocol. Machine-readable data and analysis code are available at https://github.com/Smartmoo/PABS-193-Country-Reproducibility. The numerical results can be regenerated from the same country-level dataset.

## 3 Results

### 3.1 Resource classification

**Figure 4:**
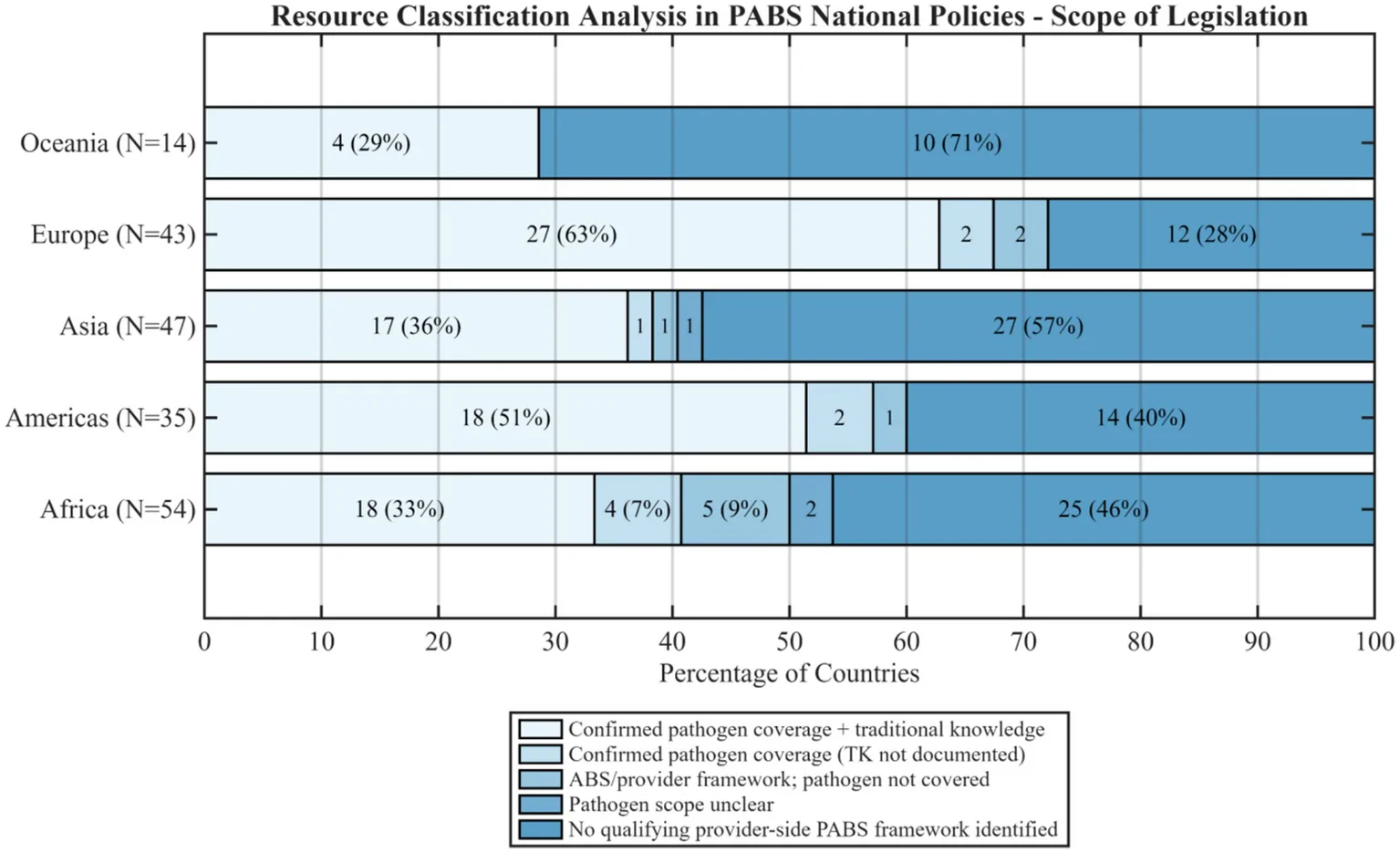
Resource classification analysis in PABS national policies-scope of legislation.

National ABS laws vary widely in scope. Many early frameworks focused on plant and animal genetic material. Broader definitions in some jurisdictions also reach microorganisms and other biological material containing functional units of heredity (55). Some systems regulate traditional knowledge associated with genetic resources as well (56). A general ABS law was not treated as evidence that pathogens were covered. Figure 4 separates confirmed pathogen coverage from broader frameworks that exclude pathogens, cases in which the scope remains unresolved and countries where no qualifying provider-side PABS framework was identified.

Figure 4 presents the overall distribution of national legal scope before the analysis turns to individual PABS components. Of the 193 UN Member States, 93 countries were confirmed to have qualifying frameworks that apply to pathogen-related genetic resources. Eighty-four of these also had documented provisions on traditional knowledge, while nine did not have such coverage documented in the analytical record. A further nine countries had a broader ABS or provider-side framework that did not cover pathogens. Three cases remained legally unclear. No qualifying provider-side PABS framework was identified in the remaining 88 countries. Together, these categories account for the full 193-country dataset.

Domestic ABS regulation and pathogen coverage are related questions, but they are not the same question. A country may regulate genetic resources generally and still exclude pathogens. Elsewhere, a broad framework exists but the available legal text does not resolve whether pathogens fall within its scope. The three uncertain cases were kept separate for that reason. Legal uncertainty can itself complicate access decisions during a health emergency (57, 58). National ABS systems also differ in the way they address traditional knowledge, a difference that is visible within the confirmed pathogen-covered group (59, 60).

The regional picture is uneven. Europe has the highest share of confirmed pathogen-covered frameworks with 29 of 43 countries, followed by the Americas with 20 of 35. Africa has 22 of 54, Asia 18 of 47 and Oceania 4 of 14. Africa also contains five countries with provider-side frameworks that do not cover pathogens and two cases with unclear scope. Asia contains one country in each of those categories. These figures describe differences in legal coverage only. They do not measure the quality or effectiveness of implementation.

Digital sequence information (DSI) is widely used in biodiversity policy, but its legal scope is not uniform. For pathogen research, relevant sequence information may derive from DNA or RNA and may circulate without the physical material. DSI was not defined in this study as DNA base pair data alone. A framework was coded as including DSI when the applicable text expressly regulated DSI or an equivalent sequence information concept. Explicit exclusion, possible inclusion and the absence of an express rule were recorded separately (61–64). Among the 93 confirmed pathogen-covered frameworks, 21 include DSI, 29 explicitly exclude it, 5 potentially include it and 38 do not address it.

**Figure 5:**
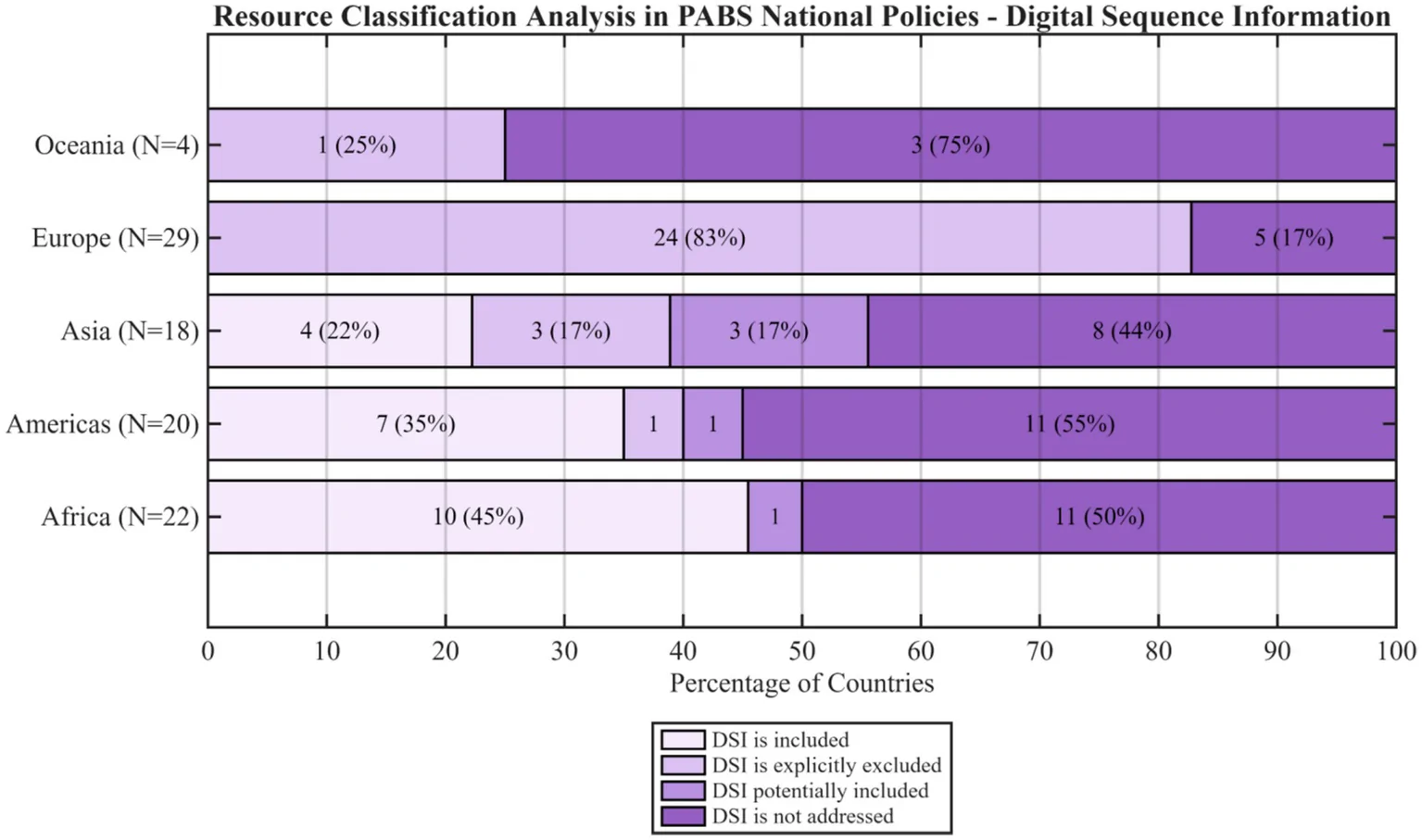
Resource classification analysis in PABS national policies-digital sequence information.

Figure 5 compares the treatment of DSI across the 93 confirmed pathogen-covered frameworks and shows clear regional differences. In Europe, 24 of 29 confirmed frameworks explicitly exclude DSI and the remaining five do not address it. Africa shows a different pattern. Ten of 22 frameworks expressly include DSI and 11 do not address it, while none was coded as an explicit exclusion. The Americas and Asia contain a mixture of inclusion, exclusion and unaddressed cases. In Oceania, three of four frameworks do not address DSI. The data indicate that the treatment of sequence information remains one of the least settled parts of national PABS governance (1, 65–67).

### 3.2 Access protocols

Domestic law determines whether access to covered genetic resources requires prior approval and what form that approval takes. CBD Article 15 recognizes national authority to regulate access, but that authority is not the same as a universal rule of State ownership over every biological resource (12, 68). In this study, access was coded as restricted when a binding condition had to be satisfied before access, such as a permit, license, consent or other authorization. It was coded as unrestricted where the applicable framework did not impose a comparable pre-access control (69).

Supplementary Figure S1 compares restricted and unrestricted access across the confirmed frameworks and shows that pre-access controls are the prevailing model. Every confirmed framework in Africa, the Americas and Oceania imposes a pre-access condition. Asia is also mainly restrictive, with 16 of 18 frameworks in this category. Europe looks different. Thirteen of its 29 confirmed frameworks restrict access and 16 are coded as unrestricted. Across the full confirmed set, 75 of 93 frameworks impose a binding pre-access condition. The remaining 18 do not. A future international PABS system will have to work with both permit based regimes and systems that do not use a comparable pre-access requirement.

Prior informed consent or an equivalent prior authorization is a common mechanism in systems that restrict access (70). The defining feature is that consent or authorization is obtained before access. Notification or registration alone was not coded as PIC. A prospective user may be asked to identify the material, its intended use and the relevant provider or institution before the competent authority or rights holder makes a decision (18, 71). Restricted access without a general PIC requirement was recorded separately.

Supplementary Figure S2 shows how PIC or an equivalent prior authorization is distributed across the confirmed frameworks. PIC is common but not universal. Sixty-one of the 93 confirmed frameworks require PIC or an equivalent prior authorization. The highest proportions occur in the Americas with 18 of 20 frameworks and Africa with 19 of 22. Asia records 12 of 18 and Oceania 2 of 4. Europe has 10 of 29, which should be read together with the 16 European frameworks coded as unrestricted. The results show that prior authorization is an important part of many national PABS systems, but the procedure is far from uniform (72).

### 3.3 Benefit-sharing

Where access is regulated, the user may also be required to enter into a contract or mutually agreed terms with the competent authority, provider or other rights holder. The instrument may be called an ABS agreement, Material Transfer Agreement or another form of written terms. It can define permitted uses, transfer conditions, reporting duties, intellectual property arrangements and monetary or non-monetary benefits (73).

Supplementary Figure S3 compares the use of contracts or comparable agreements across the five regions. Contractual requirements also vary across regions. All four confirmed frameworks in Oceania require a contract or comparable agreement. The requirement appears in 16 of 20 frameworks in the Americas, 14 of 22 in Africa and 11 of 18 in Asia. Europe again differs because 16 of 29 frameworks are unrestricted. Eleven European frameworks require a contract and two restrict access without an express contractual requirement. Overall, 56 of the 93 confirmed frameworks require a contract or comparable agreement.

Benefit-sharing may take monetary or non-monetary forms. Financial benefits can include fees, milestone payments and royalties. Non-monetary benefits may include technology transfer, research collaboration, training, information exchange or access to research results. Under the Nagoya Protocol, benefits arising from utilization are to be shared fairly and equitably on mutually agreed terms (19, 74). We coded benefit-sharing as mandatory only where the applicable framework created an enforceable obligation. General statements of equity were not treated as mandatory rules.

Supplementary Figure S4 shows how often confirmed frameworks make benefit-sharing a binding requirement. Benefit-sharing is widespread but uneven. It is required in 17 of 22 confirmed African frameworks, 16 of 20 in the Americas, 12 of 18 in Asia and 3 of 4 in Oceania. Europe records 7 of 29 because more than half of the confirmed European frameworks are coded as unrestricted. Across all 93 confirmed frameworks, 55 mandate benefit-sharing. Eighteen are unrestricted and 20 restrict access without a mandatory benefit-sharing rule identified in the coded framework.

### 3.4 Regulatory mechanism

Compliance provisions were coded separately from sanctions and from real world implementation. The relevant mechanisms include permits, certificates, checkpoints, reporting duties, record keeping and monitoring when these are expressly provided in the legal framework (75, 76). Their presence shows that a formal compliance mechanism exists in the text. It does not show how often the mechanism is used, how effectively it is enforced or how quickly it operates. For the same reason, biopiracy was not used as an automatic legal label for every breach of an ABS requirement.

Supplementary Figure S5 compares the prevalence of explicit compliance provisions across the five regions. Explicit compliance provisions are common. They appear in 88 of the 93 confirmed pathogen-covered frameworks. All confirmed frameworks in Europe, Asia and Oceania contain such provisions. They are also present in 20 of 22 African frameworks and 17 of 20 frameworks in the Americas. The remaining five cases had no explicit compliance provision identified. These figures describe the content of the legal framework. They do not indicate how consistently those rules are implemented or enforced in practice.

National laws also differ in the consequences attached to noncompliance. Depending on the jurisdiction, these may include fines, criminal liability, confiscation, restrictions on future access and the suspension or revocation of permits. Broader criminal or environmental law may apply in some cases. We recorded a sanction only when the reviewed PABS or ABS source established the relevant legal consequence (77).

Supplementary Figure S6 compares the sanction models identified in the confirmed frameworks and shows that no single model is universal. Fifty-six of the 93 confirmed frameworks combine financial penalties, criminal liability and access or administrative restrictions. Another 26 combine financial and criminal sanctions without an additional access or administrative category. Three frameworks rely on access or administrative restrictions alone, and one Asian framework combines criminal or violations liability with administrative restrictions. Seven frameworks had no explicit penalties identified. The pattern supports a comparison of legal form, not a ranking of enforcement strength.

## 4 Discussion

The mapping reveals a fragmented domestic legal landscape. Ninety-three States have confirmed frameworks covering pathogen-related genetic resources, and three cases remain legally unclear. Even within the confirmed group, rules differ in their treatment of DSI, access, PIC, contracts, benefit-sharing, compliance and sanctions. The main value of the dataset is that these differences can now be examined at country-level. It provides a baseline for considering how domestic law may interact with the emerging WHO PABS system.

Regional patterns are visible, but they should not be read as a ranking of legal maturity. Africa and the Americas rely heavily on restricted access and prior authorization. Asia is also mainly restrictive, although its treatment of DSI is mixed. Europe differs because more than half of its confirmed frameworks are coded as unrestricted and most explicitly exclude DSI. Oceania has only four confirmed frameworks, so regional percentages there should be read cautiously (78–82).

DSI marks one of the sharpest areas of divergence. Explicit exclusion is concentrated in Europe. African frameworks are divided mainly between express inclusion and the absence of a specific rule. The Americas and Asia show mixed approaches, while most Oceanian frameworks do not address DSI. Sequence information can circulate without the physical sample (83), which creates a gap between rules written for sample access and later digital use. The Cali Fund offers one example of multilateral benefit-sharing for DSI under the CBD, but it is not a pathogen specific mechanism (44, 45, 84). The PIP Framework offers a different comparison because genetic sequence data and PIP Biological Materials move through different institutional routes (24–26). The WHO negotiations will need to confront this physical and digital divide.

National access rules present a second practical challenge. Seventy-five of the 93 confirmed frameworks impose a binding pre-access condition, and 61 require PIC or an equivalent prior authorization. An international PABS mechanism will have to operate alongside those domestic requirements. Outbreak response, however, can depend on very rapid movement of samples and sequence information. Clear documentation, identifiable competent authorities and faster emergency procedures could reduce friction between these needs (85). The present data do not test whether those options would work in practice. They show where national authorization systems are likely to meet the international mechanism (86, 87).

National diversity does not require identical national rules. Countries with similar access restrictions often use different contracts, compliance mechanisms and sanctions. PABS also intersects with intellectual property, public health law, environmental regulation, biosafety and research governance (88, 89). A more workable objective is interoperability in a limited set of functions. States need to know which authority grants access, what evidence of authorization is required, how material or sequence use is documented and how benefit obligations are communicated. Political differences will remain. Provider States, researchers, manufacturers, communities and public health authorities do not always place the same weight on speed, sovereignty, equity, legal certainty and access to countermeasures. Rules that are too prescriptive may be difficult to accept. Rules that are too loose may leave the system fragmented.

These differences matter for the PABS system under the WHO Pandemic Agreement. National starting points vary across DSI, authorization, contracts, benefit-sharing, compliance and sanctions. The Annex was still under negotiation at the study cutoff, so there is little basis for assuming that one domestic model will become the international standard (36–38). The Cali Fund shows that multilateral benefit-sharing for DSI can operate under another international regime, although its design cannot simply be transferred to pathogens (44, 45, 84). The policy literature has also discussed faster emergency procedures, predefined benefit options and stronger capacity support (90). This study makes a narrower contribution. It identifies the domestic legal variation that any of these choices would have to accommodate.

## 5 Conclusion

The global PABS landscape remains legally diverse. This study identified national frameworks with provisions applicable to pathogen-related genetic resources in 93 UN Member States. The content of these frameworks varies considerably with respect to digital sequence information, access requirements, prior authorization, contractual arrangements, benefit-sharing, compliance and sanctions. These differences matter because any future WHO PABS system will need to interact with existing national legal environments rather than operate independently of them.

A central policy challenge is therefore to connect the rapid sharing of pathogens and related sequence information with benefit-sharing arrangements that are credible to providers, predictable for users and workable during public health emergencies. Clearer rules concerning authorization, documentation, sequence information and benefit obligations may help reduce legal and operational uncertainty. Capacity building, technical cooperation and implementation support may also be important in some settings, although the present study maps legal and policy frameworks rather than measuring national implementation capacity. The evidence therefore points toward greater interoperability among national systems rather than toward uniformity in domestic legal models.

The policy mapping further highlights several considerations relevant to the future development of PABS arrangements. First, national sovereignty over genetic resources and the need for timely international cooperation in global health should be accommodated within the same institutional framework rather than treated as mutually exclusive objectives. Second, the increasing importance of sequence information means that governance arrangements cannot rely exclusively on approaches developed for the transfer of physical genetic materials. Future rules will need to address the distinctive characteristics of sequence information while preserving the objective of fair and equitable benefit-sharing. Third, the diversity and uneven development of national legal frameworks suggest that implementation support, capacity building and technical assistance may need to accompany international rules, particularly where domestic institutional arrangements remain limited. Fourth, the considerable variation identified across national systems suggests that international rules may need to combine common principles and minimum requirements with sufficient flexibility for implementation in different domestic legal and institutional contexts.

The WHO Pandemic Agreement provides a common institutional framework for addressing these issues, while the detailed PABS arrangements remain under negotiation through the Annex described in Article 12 (36–38). As of August 2026, negotiations had not been concluded, and the eighth meeting of the IGWG was scheduled for 14–18 September 2026. The country-level mapping developed in this study therefore provides a baseline for assessing how the eventual international rules may interact with existing domestic law. Its principal contribution is descriptive and comparative. It identifies shared features as well as legal differences that may affect future access to and sharing of pathogens and related sequence information. The experience of COVID-19 underscored both the importance of timely pathogen sharing and persistent inequities in access to vaccines, diagnostics and other health products. Future PABS arrangements will therefore need to address timely access and equitable benefit-sharing together, while remaining sufficiently compatible with the diverse national legal frameworks identified in this study.

## Data Availability

The original contributions presented in the study are included in the article/Supplementary material, further inquiries can be directed to the corresponding author.
