# Supplementary material for: Navigating the policy landscape: a systematic mapping review of national access and benefit-sharing frameworks for pathogens
Source: Front Public Health. 2026 Sep 4;14:1908687. doi: 10.3389/fpubh.2026.1908687 (PMC13585930; doi:10.3389/fpubh.2026.1908687)
Supplement: Supplementary file 1 [file Supplementary_file_1.docx]

**Supplementary Materials:**

Supplementary Dataset S1 contains the 193-country legal corpus and final codes. Supplementary Table S2 records country-level search, screening, exclusion and uncertainty outcomes. Supplementary Methods S3 provides the coding manual and search protocol. Supplementary Figures S1-S6 provide the detailed regional distributions for access to resources, prior informed consent, contractual terms, benefit-sharing, compliance provisions, and legal sanctions. The supplementary files and machine-readable analysis code are available at <https://github.com/Smartmoo/PABS-193-Country-Reproducibility.>

Figure S1. Resource Classification Analysis in PABS National Policies-Access to Resources

Figure S2. Resource Classification Analysis in PABS National Policies-Prior Informed Consent

Figure S3. Resource Classification Analysis in PABS National Policies-Contractual Terms

Figure S4. Resource Classification Analysis in PABS National Policies-Benefit-sharing

Figure S5. Resource Classification Analysis in PABS National Policies-Compliance

Figure S6. Resource Classification Analysis in PABS National Policies-Legal Sanctions
